# Risk of second revision and mortality following first-time revision due to prosthetic joint infection after primary total hip arthroplasty: results on 1,669 patients from the Danish Hip Arthroplasty Register

**DOI:** 10.2340/17453674.2024.41913

**Published:** 2024-09-13

**Authors:** Rajzan JOANROY, Sophie GUBBELS, Jens K MØLLER, Søren OVERGAARD, Claus VARNUM

**Affiliations:** 1 Department of Orthopaedic Surgery, Lillebaelt Hospital – Vejle; 2 Department of Regional Health Research, University of Southern Denmark; 3 Division of Infectious Disease Preparedness, Statens Serum Institut; 4 Department of Clinical Microbiology, Lillebaelt Hospital – Vejle; 5 Department of Orthopedic Surgery and Traumatology, Copenhagen University Hospital, Bispebjerg; 6 Department of Clinical Medicine, Faculty of Health and Medical Sciences, University of Copenhagen, Denmark

## Abstract

**Background and purpose:**

Prosthetic joint infection (PJI) following total hip arthroplasty (THA) has a severe impact on patients. We investigated the risk of second revision and mortality following first-time revision due to PJI.

**Methods:**

We identified 1,669 first-time revisions including 416 treated with debridement, antibiotics, and implant retention (DAIR) from the Danish Hip Arthroplasty Register (DHR). First-time revision due to PJI was defined as a revision with ≥ 2 culture-positive biopsies for the same bacteria or re-ported as PJI to the DHR within 1 year after primary THA with non-PJI revisions as controls. We retrieved information on Charlson Comorbidity Index (CCI), death, cohabitation status, and cultures from intraoperative biopsies. The adjusted relative risk (RR) with 95% confidence interval (CI) was calculated by first-time revision (PJI or non-PJI). Patients were followed from first-time revision until end of study.

**Results:**

PJI was found in 140 of 280 patients having a second revision following any first-time revision. Of these 280 patients, 200 were treated with DAIR as second revision. Patients with first-time revision due to PJI had an increased risk of second revision compared with first-time revision for non-PJI with an adjusted RR for second revision due to any cause of 2.7 (CI 1.9–3.8) and second revision due to PJI of 6.3 (CI 4.0–10). The 10-year adjusted RR for mortality for patients with first-time revision due to PJI compared with non-PJI was 1.8 (CI 0.7–4.5).

**Conclusion:**

The risk of second revision was increased both for second revision due to any reason and due to PJI following first-time revision due to PJI. Mortality risk following first-time revision due to PJI was increased, but not statistically significant.

The most devastating and feared complication related to primary total hip arthroplasty (THA) is prosthetic joint infection (PJI) requiring revision, because of its association with pain and suffering, increased hospital stay, more readmissions, subsequent revisions, increased morbidity, increased mortality, and costs [1,2].

When a PJI occurs, most patients undergo revision surgery [3]. However, studies on prognosis after first revision due to PJI are very sparse regarding the risk of second revision. A recent study investigated the outcome after first-time revision due to PJI and found a rate of second revision of 27% and a mortality rate of 8% at 1-year follow up [4]. However, these authors compared different surgical strategies (debridement, antibiotics and implant retention, 1-stage revision, and 2-stage revision). Furthermore, it was based on a single center with a relatively small sample size of 369 patients.

Another study found a significantly increased mortality risk after 1 year following first-time revision due to PJI compared with first-time revision due to aseptic cause [5]. However, the risk of second revision was not studied. Studies on risk of revision and mortality should preferably be based on large cohorts from national registers with high completeness and systematic follow-up.

We primarily aimed to study the risk of second revision due to any cause for patients who underwent first-time revision due to PJI compared with revision due to non-PJI within 1 year after a primary THA by utilizing large Danish health registers. The secondary outcomes were second revision due to PJI and mortality risk up to 10 years following first-time revision.

## Methods

This was a population-based cohort study conducted in Denmark utilizing nationwide longitudinally maintained data from Danish health registers. The unique 10-digit identification number assigned to all Danish citizens was used to link data between registers on an individual level. We report this study according to the Reporting of studies Conducted using Observational Routinely-collected Data (RECORD) guidelines.

### Data sources

*The Danish Hip Arthroplasty Register (DHR).* The DHR is a clinical quality database that contains information on primary THA and revisions in Denmark. It is mandatory for all public and private hospitals in Denmark to report to the DHR [6]. The registration completeness was 97% for primary THA and 90% for revision at the end of the study period [7]. Both the diagnosis of primary THA and PJI have been validated [8,9]. The DHR includes data on surgery date, surgery type, indication for surgery, femoral head size, duration of surgery, type of operation theatre, and duration of antibiotic treatment.

*The Danish National Patient Register (DNPR).* This register holds information on all discharge diagnoses and date of discharge since 1977, since 1994 according to the International Classification of Diseases 10th edition [10]. The Charlson Comorbidity Index (CCI) score was determined at the time of surgery using data from the DNPR with validated diagnoses up to 10 years prior to surgery [11]. CCI was categorized into 3 levels of comorbidity: CCI score of 0 (low); CCI score of 1–2 (medium); and CCI score of ≥ 3 (high) [12].

*The Civil Registration System (CRS).* The CRS holds information on date of birth, sex, emigration status, and date of death. This register provides complete follow-up data, allowing for comprehensive patient tracking [13].

Statistics Denmark. Statistic Denmark contains information on socioeconomic characteristics. As cohabitation status has been associated with increased risk of revision following primary THA [14] this register was used to divide patients into living alone or cohabitating.

*Healthcare-Associated Infections Database (HAIBA).* HAIBA, an automated surveillance system, registers certain infections acquired in Danish hospitals [15,16]. HAIBA utilizes data from the Danish Microbiological Database and DNPR and monitors first-time revision within 1 year after primary THA based on microorganisms cultured from intraoperative biopsies taken during revision and analyzed at departments of clinical microbiology in Denmark. HAIBA defines a revision to be due to PJI if performed within 1 year after primary THA with ≥ 2 biopsies growing the same bacteria of at least 3 biopsies taken.

### Study population

All revisions registered in the DHR during 2010–2019 were retrieved. This included debridement, antibiotics and implant retention (DAIR) with or without exchange of removable parts (i.e., liner and/or head) and complete or partial removal or exchange of implant components. 2-stage revisions comprising several procedures (implant removal and reimplantation) were considered as 1 operation. The inclusion criterion was first-time revision within 1 year after primary THA, which allowed for linkage of microbiological data via HAIBA. In cases of bilateral THAs with bilateral revision, the hip with the first revision was included and followed. Revisions that occurred more than 1 year after primary THA or lacked information on duration of surgery, duration of antibiotic treatment, indication for revision, and cohabitation status were excluded. This allowed for complete follow-up of the study population, and all patients were followed for at least 1 year after the first-time revision or until the occurrence of second revision, death, emigration, or end of study (December 31, 2019), whichever came first. First-time revision due to PJI was defined as a revision with ≥ 2 culture-positive biopsies for the same bacteria or a reported PJI in the DHR regardless of later results of cultures, thus complying with the widely accepted definition of confirmed PJI in regards to clinical features and intraoperative biopsies [17]. Revisions due to causes other than PJI were defined as revisions due to non-PJI and used as controls.

### Outcomes

The primary outcome was a second revision due to any cause up to 10 years following first-time revision. A second revision included DAIR with or without exchange of removable parts (i.e., liner and/or head), complete or partial removal, or exchange of implant components. The secondary outcomes were second revision due to PJI and the risk of mortality up to 10 years following first-time revision. Additionally, we reported on other specific causes of second revision: fracture, dislocation, pain and “other.” The classification of second revision was based on the reported indication in the DHR, since HAIBA does not include revisions performed more than 1 year after the primary THA.

### Statistics

The characteristics of the patients were reported as counts and percentages for the included variables, except for age and duration of surgery, which is given as median with interquartile range (IQR) due to skewness tested visually with a histogram.

Causes of second revision are reported as counts with percentages of the total in the study population. The cumulative incidence of second revision for the study population is reported with 95% confidence intervals (CI) with death as competing risk [18]. The relative risk (RR) of second revision was estimated with the pseudo-value method using generalized linear regression and reported with 95% CI [19]. The pseudo-value method transforms the data and creates pseudo observations at pre-specified time points [20]. This method is appropriate for survival analysis with censored data, which allows for regression analysis as if there is no censoring in time-to-event. The stset, stpci, glm pseudo with fam(gauss) link(log) vce(robust), and eform were used in STATA. We adjusted for the covariates related to the patient characteristics and surgery (see Appendix Figure 1): sex, age groups (< 65, 65–74, ≥ 75), CCI (low, medium, high), cohabitation status (cohabiting or living alone), femoral head size (< 36, 36, > 36, missing), antibiotic treatment in relation to first-time revision (only preoperatively, maximum 24 hours, > 24 hours). For the adjusted RR estimations, we allowed for 1 degree of freedom for every 10 outcome events, thus reducing the risk of overfitting the models. All outcomes were evaluated at the end of the study resulting in a potential follow-up time of up to 10 years.

**Figure 1:**
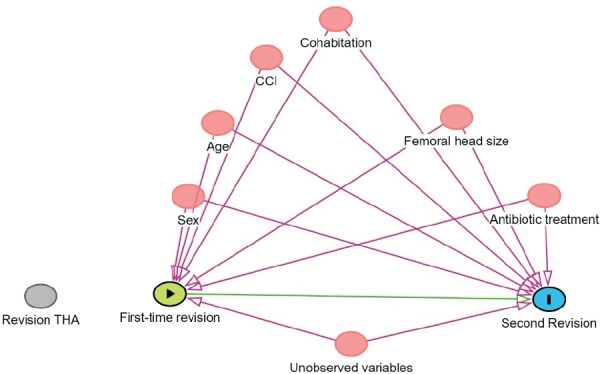
DAG demonstrating variables considered as confounders in causal pathways.

Sensitivity analyses were performed for the primary outcome of any second revision. We differentiated between first-time revision due to PJI treated with DAIR and first-time revision due to PJI treated with complete revision (1-stage and 2-stage) with first-time revision due to non-PJI as control group.

For the statistical analyses and graphs, we used STATA version 17.0 (StataCorp LLC, College Station, TX, USA).

### Ethics, funding, data sharing, use of AI, and disclosures

This study was designed as non-interventional, and approval from an ethics committee was not required. The Danish Regional Internal Directory has approved this study (journal number 20/7287). The study did not receive any specific grant from funding agencies in the public, commercial, or not-for-profit sectors. If reasonably requested to the corresponding author, the metadata for this study can be provided. AI was not used for this study. The authors declare no conflicts of interest in relation to this study. Complete disclosure of interest forms according to ICMJE are available on the article page, doi: 10.2340/17453674.2024.41913

## Results

**Figure 2:**
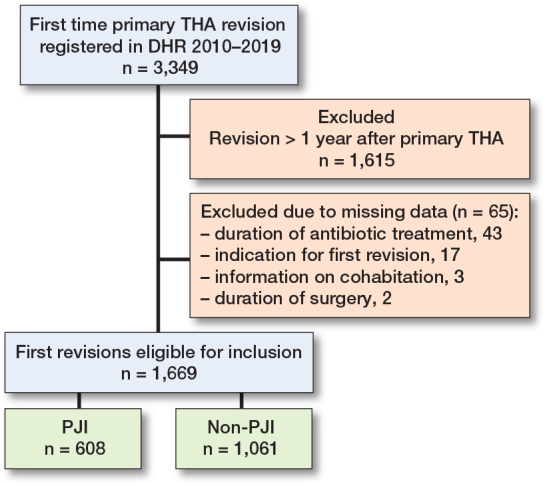
Flowchart of the study population. DHR = Danish Hip Arthroplasty Register; THA = total hip arthroplasty.

We extracted 3,349 first-time revisions from the DHR during 2010–2019 and excluded 1,680 by the exclusion criteria. This resulted in a cohort of 1,669 first-time revisions eligible for statistical analysis (Figure 2). 68% of the 1,669 patients had registered biopsies taken during first-time revision. In 97 cases (6%), the cause of first-time revision was corrected from non-PJI to PJI if ≥ 2 biopsies were culture-positive for the same bacteria out of ≥ 3 taken biopsies. This was done to increase the probability of identifying PJIs more correctly, since PJI is underreported in the DHR [21]. 608 patients underwent first-time revision due to PJI, while 1,061 had first-time revision due to non-PJI causes. Of the 608 first-time revisions due to PJI, there were 416 (69%) who were treated with DAIR as the first-time revision. For staged revisions, the average interim period between implant removal and reimplantation was 115 days. The median age was 71 (IQR 64–77) for both groups, and the majority of the study population were females (55%). The distributions of CCI, cohabitation status, and type of operation theatre were similar in both groups. However, the first-time revisions due to PJI had longer duration of surgery and higher percentage of antibiotic treatment exceeding 24 hours (Table 1).

**Table 1:** Demographics of the study population by first-time revision due to prosthetic joint infection (PJI) or non-PJI revision following total hip arthroplasty. Values are count (percentage column wise) unless otherwise specified

Factor	PJI revision (n = 608)	Non-PJI revision (n = 1,061)
Age groups		
≤ 64	158 (26)	283 (27)
65–74	251 (41)	411 (39)
≥ 75	199 (33)	367 (34)
Sex		
Female	295 (49)	617 (58)
Male	313 (51)	444 (42)
Charlson Comorbidity Score		
Low 0	334 (55)	568 (53)
Medium 1–2	207 (34)	358 (34)
High ≥ 3	67 (11)	135 (13)
Cohabitation status		
Alone	249 (41)	464 (44)
Cohabitant	359 (59)	597 (56)
Head size in mm, n		
< 36	190 (31)	507 (48)
36	325 (53)	490 (46)
> 36	8 (1.3)	30 (2.8)
Missing	85 (14)	34 (3.2)
Minutes of first-time revision^a^	80 (60–112)	75 (59–100)
Operation theatre		
Laminar flow	542 (89)	915 (86)
Conventional	66 (11)	146 (14)
Duration of antibiotic treatment in relation to revision		
Only preoperatively	2 (0.3)	30 (2.8)
Max 24 hours	47 (7.7)	361 (34)
> 24 hours	559 (92)	670 (63)

a Median with interquartile range (IQR).

### Risk of second revision

280 patients underwent a second revision due to any cause, with the most prevalent cause being PJI, accounting for 140 revisions (Table 2). Of the 280 patients who underwent a second revision due to any cause, 200 were treated with DAIR at the second revision.

**Table 2:** 280 had a new revision distributed into causes of second revision by first-time revision due to prosthetic joint infection (PJI) or non-PJI revision following total hip arthroplasty. Values are count (% of total number of patients in each specific group)

Second revision due to	First-time revision due to	
	PJI n = 608	non-PJI n = 1,061
PJI	108 (18)	32 (3.0)
Dislocation	8 (1.3)	48 (4.5)
Femoral fracture	5 (0.8)	12 (1.1)
Aseptic loosening	12 (1.9)	27 (2.5)
Pain without loosening	0 (0)	6 (0.6)
Other	5 (0.8)	17 (1.6)
Total	138 (23)	142 (13)

For second revision due to any cause, the cumulative 10-year incidence was 64% (CI 52–74) and 56% (CI 45–65) for first revision due to PJI and non-PJI, respectively.

**Figure 3:**
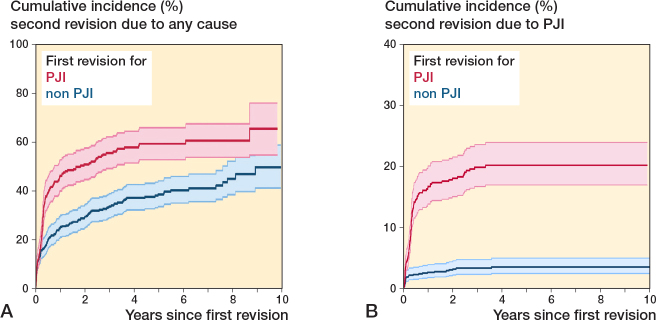
Cumulative incidence for second revision due to any cause (A) and due to prosthetic joint infection (PJI) (B) at the end of study by cause of first revision.

For first-time revision due to PJI, the cumulative incidence of a second revision due to PJI was 19% (CI 16–22) and for first-time revision due to non-PJI it was 3% (CI 2–5) (Figure 3 A and B). The risk of a second revision due to any cause was significantly higher for patients who underwent first-time revision due to PJI compared with non-PJI with adjusted RR 2.7 (CI 1.9–3.8). The same was even more evident for a second revision due to PJI when comparing first-time revision due to PJI with non-PJI with adjusted RR 6.3 (CI 4–10). There was no difference in the risk of second revision due to fracture, pain and “other,” but a lower risk of dislocation may be present for patients who underwent first-time revision due to PJI compared with non-PJI (Table 3).

**Table 3:** Relative risk (RR) of second revision with 95% confidence interval (CI) for first-time revision due to prosthetic joint infection (PJI) with first-time revision due to non-PJI as reference

Second revisions	n	Crude RR (CI)	Adjusted RR (CI)
Any revision	280	2.1 (1.6–2.7)	2.7 (1.9–3.8) ^a^
PJI revision	140	6.3 (4.1–9.5)	6.3 (4.0–10) ^b^
Fracture	17	0.9 (0.3–3.0)	
Dislocation	56	0.2 (0.1–0.5)	0.2 (0.1–0.6) ^c^
Pain	6	0.0 (0.0–0.1)	
Other	22	1.0 (0.3–2.9)	0.8 (0.1–7.3) ^d^

a Adjusted for: sex, age groups, Charlson comorbidity index, cohabitation status, femoral head size and duration of antibiotic treatment in relation to revision.

b Adjusted for: sex, age groups, Charlson Comorbidity Index, cohabitation status.

c Adjusted for: sex, age groups.

d Adjusted for: sex.

The sensitivity analyses showed that the adjusted RR of any second revision for cases of first-time revision due to PJI treated with DAIR compared with first-time revision due to non-PJI was 1.3 (CI 0.7–2.2). For cases of first-time revision due to PJI treated with complete revision (1-stage or 2-stage) compared with first-time revision due to non-PJI, the adjusted RR of any second revision was 3.0 (CI 2.1–4.3).

### Mortality risk

**Figure 4:**
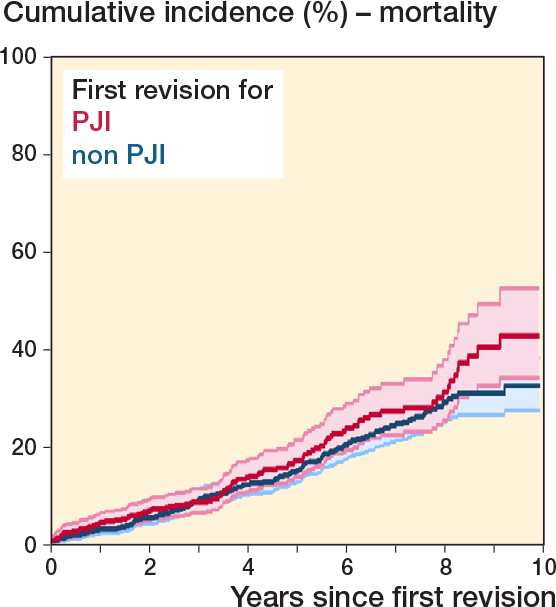
Survival probability following first revision with mortality as endpoint.

During the study period, 287 (17% of the population) died following any first-time revision. For first-time revision due to PJI, the cumulative mortality at 10 years’ follow-up was 43% (CI 34–52) and for non-PJI revisions 33% (CI 28–38) (Figure 4). The crude and adjusted 10-year mortality risk for patients undergoing first-time revision due to PJI compared with non-PJI revisions was 1.4 (CI 0.8–2.3) and 1.8 (CI 0.7–4.5), respectively.

## Discussion

We aimed to study the risk of second revision for patients who underwent first-time revision due to PJI compared with revision due to non-PJI within 1 year after a primary THA. In this national population-based register study utilizing data from Danish health registers, we showed that patients who underwent first-time revision due to PJI, compared with those who underwent first-time revision for non-PJI, had a statistically significantly higher risk of undergoing a second revision due to any cause and specifically a second revision due to PJI. However, the risk of mortality for patients who underwent first-time revision due to PJI compared with those who underwent revision due to non-PJI was not increased within 10 years.

A recent systematic review reported infection rates of 10% up to 45% following first-time revision due to PJI, but only DAIR were included [22]. Another study reported on the outcomes following first-time revision due to PJI and found a second revision rate of 26%; however, this was based on a relatively small cohort [23]. It has been established that patients who undergo revision surgery for PJI have an increased risk of unplanned hospital readmissions and an extended hospitalization period, underscoring the significant effect it exerts on patients’ well-being [1,2]. However, none of the published studies has directly investigated the association between first-time revision due to PJI and the risk of second revision. Lenguerrand et al. found an overall second-revision risk of 15% following first-time revision due to PJI, but their primary aim was to compare 1-stage with 2-stage revision [24]. Other studies have investigated the risk of mortality and found it to be significantly higher in patients who underwent first-time revision due to PJI [5,25]. However, the study by Zmistowski et al. was based on a single-center dataset, and Gundtoft et al. limited their mortality risk follow-up to 1 year after first-time revision due to PJI. In contrast, some studies have found no association between first-time revision due to PJI following primary THA and the mortality risk [26,27]. However, these studies are small sample cohort studies from single centers with 134 and 186 patients included, respectively.

Our results indicate that a reduced risk of second revision due to dislocation may be present following first-time revision due to PJI. A possible explanation could be that first-time revision due to non-PJI might be major aseptic revisions leading to higher dislocation rates. Another possible explanation of this result could be that the majority of second revisions are performed in the early period following first-time revision (Figure 3A), indicating that those patients with first-time revision due to PJI undergo second revision before the occurrence of a potential dislocation.

### Strengths and limitations

Because of the comprehensive Danish registers, we were able to include a substantial study population, which is important when investigating rare long-term risk associations. Through high completeness of data from DHR and CRS, we maintained complete follow-up on all patients with a negligible risk of death misclassification, estimated to be close to 0% [13]. The comprehensive follow-up greatly minimizes the risk of selection bias. Additionally, the utilization of microbiological data from intraoperative biopsies increased the sensitivity of the validated PJI diagnosis up to 90% [8]. Moreover, it aligns our study with the widely accepted definition criterion for PJI, which requires ≥ 2 culture-positive biopsies for the same bacteria or PJI reported by the surgeon [17].

The sensitivity analysis indicated that the cases of first-time revision due to PJI treated with DAIR did not have an increased risk of second revision compared with first-time revision due to non-PJI, and the cumulative incidence of outcome following first-time revision due to PJI treated with DAIR seems to agree with the existing literature [28]. The results may indicate that first-time PJIs treated with complete revisions are more severe cases than those treated with DAIR.

This study is not without its limitations. First, even though we have used microbiological data, there remains a slight risk of misclassifying PJI revision as non-PJI, as biopsy results were missing in 32% of the primary revisions. This could potentially lead to underestimations in both the incidence of PJI and the associated mortality risk. Second, key health indicators such as smoking status, obesity, and alcohol consumption, which can impact the risk of revision due to PJI and mortality, are not reported to the DHR. These factors may introduce unmeasured confounding; however, we were able to adjust for the CCI. Third, our dataset was constrained, preventing the inclusion of first-time revision occurring more than 1 year after primary THA. However, most PJIs occur within the first year (cumulative incidence of 0.86%) compared with PJIs identified within 5 years (cumulative incidence of 1.03%) after the primary THA [21].

### Conclusion

We found a statistically significantly increased risk of any second revision and second revision due to PJI following a first-time revision due to PJI compared with non-PJI. However, the occurrence of a first-time revision due to PJI did not increase the risk of mortality within a 10-year period after primary THA. First-time revision due to PJI treated with DAIR does not seem to have an increased risk of second revision compared with first-time revision due to non-PJI.

## Notes

All authors contributed significantly to conceptualization, methodology, writing–review, and editing. RJ and CV conducted formal analyses. RJ conducted data management and the original draft. All authors contributed to interpreting the results, revising and discussing the manuscript.

The authors would like to thank the Danish Clinical Quality Program – National Clinical Registries for data extraction from the DHR and Odense Patient data Explorative Network (OPEN) for their help and advice.

Handling co-editors: Bart A. Swierstra and Philippe Wagner

*Acta* thanks Marc Nijhof and Ewout S. Veltman for help with peer review of this manuscript.
